# More counselling for end-of-life decisions by GPs with own advance directives: A postal survey among German general practitioners

**DOI:** 10.1080/13814788.2017.1421938

**Published:** 2018-03-16

**Authors:** Rieke Schnakenberg, Lukas Radbruch, Christine Kersting, Friederike Frank, Stefan Wilm, Denise Becka, Klaus Weckbecker, Markus Bleckwenn, Johannes M. Just, Michael Pentzek, Birgitta Weltermann

**Affiliations:** a Institute of General Practice and Family Medicine, Medical Faculty, University of Bonn, Bonn, Germany;; b Department of Palliative Medicine, University Hospital Bonn, Bonn, Germany;; c General Medicine, Teaching Area RWTH Aachen University, Aachen, Germany;; d Institute of General Practice, Medical Faculty, Heinrich Heine University Düsseldorf, Düsseldorf, Germany;; e Department of General Practice, Medical Faculty, Ruhr University of Bochum, Bochum, Germany;; f Institute for General Medicine, University of Duisburg-Essen, Essen, Germany

**Keywords:** Medical consultation, general practice/family medicine, advance directive/living will, cross-sectional survey

## Abstract

**Background:** Although general practitioners (GPs) are among the preferred contact persons for discussing end-of-life issues including advance directives (ADs), there is little data on how GPs manage such consultations.

**Objectives:** This postal survey asked German GPs about their counselling for end-of-life decisions.

**Methods:** In 2015, a two-sided questionnaire was mailed to 959 GPs. GPs were asked for details of their consultations on ADs: frequency, duration, template use, and whether they have own ADs. Statistical analysis evaluated physician characteristics associated with an above-average number of consultations on AD.

**Results:** The participation rate was 50.3% (*n* = 482), 70.5% of the GPs were male; the average age was 54 years. GPs had an average of 18 years of professional experience, and 61.4% serve more than 900 patients per three months. Most (96.9%) GPs perform consultations on living wills (LW) and/or powers of attorney (PA), mainly in selected patients (72.3%). More than 20 consultations each on LWs and PAs are performed by 60% and 50% of GPs, respectively. The estimated mean duration of consultations was 21 min for LWs and 16 min for PAs. Predefined templates were used in 72% of the GPs, 50% of GPs had their ADs. A statistical model showed that GPs with ADs and/or a qualification in palliative medicine were more likely to counsel ≥20 patients per year for each document.

**Conclusion:** The study confirmed that nearly all German GPs surveyed provide counselling on ADs. Physicians with ADs counsel more frequently than those without such documents.

KEY MESSAGES

- The mail survey yielded a high response rate (50.3%).
- Integration into regular care is a barrier: only 27.7% think that such consultations can easily be integrated into office hours under current conditions.
- GPs with ADs and/or additional training reported significantly higher consultation rates.

## Introduction

Patient autonomy is recognized as one of the most fundamental principles in medical ethics, and a particular area of concern is how autonomy can be maintained for patients with a severely impaired decision-making capacity [1]. Advanced care planning (ACP) with the timely discussion of end-of-life issues has become an increasingly important issue in all Western nations [2–5]. Within the scope of country-specific societal discussions, legal regulations in many countries support the documentation of patients’ advance directives (AD) in the form of living wills (LWs) and/or powers of attorney (PA) for healthcare [6]. While a LW documents personal preferences and wishes for future medical care including its cessation, a PA authorizes one or more specific persons as surrogate decision makers in case of lost capacity [1,7,8]. As recommended before, information is a core issue and has to be provided in accordance with the different causes and different circumstances when establishing ADs [9].

Despite these developments, the prevalence of ADs is low; in Belgium and the Netherlands, for example, 2–3% of persons aged below 60, about 10% of those aged ≥60, and up to 23% of relatives of decedents have ADs [10,11]. In Germany, the prevalence of ADs in the general population was between 2.5 and 10%, while a survey of GP patients ≥85 years reported that 69% have an AD and 64.6% a PA [12,13]. Beside notaries, general practitioners (GPs) are the preferred contact persons for discussing end-of-life issues and preparing such documents [14,15], yet little research has been conducted in primary care settings [16]. Two studies from Canada and the US showed that patients who consider ADs important prefer their GP to start the conversation about these documents [15,17]. GP’s consultations were shown to be more effective at increasing AD completion than passive education by written materials [6]. To enable individuals to make an informed decision, more counselling for the public and patients as well as education for health professionals are considered important [17].

Since the introduction of new laws in Germany in 2009 and 2015, the interest in ADs and ACP has been strengthened [2,5]. The German Act to improve hospice and palliative care from 2015 specified that nursing homes are obliged to offer ACP to their residents, but this is not yet routinely established [5]. Although GPs are confronted regularly with terminally ill patients and their palliative care, there is little data on how German GPs manage consultations on end-of-life issues including the preparation of LWs and/or PAs. This postal survey among GPs in North-Rhine Westphalia addressed the duration, frequency, and target groups for such consultations, and whether physicians with and without own ADs differ regarding their counselling behaviour.

## Methods

### Study design

In April and May 2015, a two-page questionnaire was mailed to 959 GPs in academic teaching practices from eight universities in North-Rhine Westphalia (NRW) together with a pre-stamped return envelope. The GPs were recruited by the university institutes for general practice of these universities (Aachen, Bochum, Bonn, Cologne, Düsseldorf, Essen, Münster, Witten-Herdecke), which collaborate in the regional family medicine competence network (Kompetenzverbund Allgemeinmedizin NRW). To enhance study participation, the questionnaire was limited to two pages and a small package of candies was offered as an incentive. The anonymous survey received ethical approval from the Ethics Committee of the Medical Faculty of the University of Duisburg-Essen (No. 15-6247-BO).

### Questionnaire: definitions and items

Researchers of the Kompetenzverbund Allgemeinmedizin NRW developed the questionnaire. The two-sided questionnaire along with a cover letter described the project and defined the terms LW and PA:

- Living will (LW) was defined as a document on the patient’s wishes regarding medical treatment in case the capacity to give consent is lost.
- Power of attorney (PA) was defined as a document which authorizes one or more persons to execute comprehensive or specific tasks if the principal is incapable of handling these tasks.

The questionnaire was pretested by four GPs with teaching obligations and by two researchers in the field. In the pretest, we used cognitive interviewing techniques to study comprehensibility as well as the order and the content of the various items.

The following questions were asked addressing the consultation characteristics separately for LWs and PAs:

- How often do you provide consultations on advance directives? (5-point Likert scale from ‘never’ up to ‘>20 times per year’)
- How long do your consultations on living will/power of attorney last on average (in minutes)?
- Do you use a predefined template for LW/POA? (yes/no)
- If yes, which source of origin do you use? (free text)
- How often do patients bring predefined templates in? (5-point Likert scale from ‘occasionally’ up to ‘very often’)
- How do you file LW/AD documents? (‘note,’ ‘scan,’ ‘copy,’ ’not at all,’ ‘other’)
- Do you offer consultations on both documents simultaneously? (5-point Likert scale from ‘yes, always’ up to ‘no, never’)
- What fee (euro) do you consider appropriate for a 30-min consultation on AD? (free text)
- Do you have prepared a personal LW/PA (yes/no), and if not, why (free text)?

Additionally, sociodemographic characteristics were requested: age, gender, specialization (in Germany, a GP can be qualified as a general internist and/or family physician), whether they completed a 40-h training on palliative care, and whether they hold a subspecialty certificate in palliative medicine; in addition, practice characteristics were requested: location (answer options: city with >100,000 inhabitants, city with <100,000 inhabitants, rural region), number of physicians working in the practice (salaried doctors and practice owners), estimated mean number of patients per physician in three months (options: > 700, about 800, > 900), and years of professional experience.

### Statistical analysis

The participation rate was calculated as the percentage of returned questionnaires compared to those dispatched. All questionnaires returned were included in the analysis. Frequencies were calculated for categorical data; continuous data are provided as the mean with standard deviation (SD). Percentages and mean values are reported for valid cases. Subpopulations were compared regarding their sociodemographic and consultation characteristics: (a) GPs with and without ADs (LW and/or PA), and (b) GPs with and without an above-average number of consultations on LWs as well as PAs per year (cut-off: median for the study population).

All categorical variables regarding associations between physician and consultation characteristics, which were significant (level of *p* <.05) in initial bivariate analyses (chi-square statistics) were included in the final logistic regression models. These addressed the association between physician characteristics and an above-average number of consultations both for LWs and PAs per year and the association between physician characteristics and having their own AD. Statistical significance was assigned at the level of *p* <.05. The statistical analysis was performed using IBM SPSS Statistics, Version 24.

## Results

### Study population

A total of 482 of 959 physicians answered the survey, yielding a response rate of 50.3%. The average age of the respondents was 53.7 years (SD: 7.6; range: 33–72), 70.5% were male, and 45.3% practice in an urban area. More than half of the GPs (53.4%) had completed a 40-h training in palliative care. Of the respondents, 50% had prepared their ADs, i.e. LW and/or PA: 44.8% had their LW, 42.9% had a PA. Of those without ADs, 77% provided a reason for not completing these documents: suppression/neglect (18.5%), current age (14.1%), and lack of time (13.7%). Few physicians (6%) stated negative reasons (‘not useful,’ ‘I refuse AD’). See Table 1 for details on physician characteristics.

**Table 1.** Characteristics of participating GPs (*n* = 482).

Characteristics of physicians	Percent for categorical variables; mean (SD, min, max) for continuous variables
Male	70.5
Mean age, years (SD, min, max)	53.8 (7.6, 33, 72)
Board certificate in:	
General medicine	75.9
Internal medicine	17.1
Both	7.0
Mean years of professional experience (SD, min, max)	18.1 (8.7, 1, 46)
Group practice	69.6
Number of physicians in practice (SD, min, max)	2.3 (1.3, 1, 10)
Practice region:	
Urban area (city >100,000 inhabitants)	45.3
City or rural area (<100,000 inhabitants)	54.6
Number of patients per three months:	
<900	38.6
> = 900	61.4
Additional training in palliative medicine:	
Completed 40-hour course in palliative medicine	53.4
Additional sub-specialization degree in palliative medicine	17.7
Has own advance directives:	
Has living will	44.8
Has power of attorney for healthcare	42.9
Has both documents	37.8
Has living will and/or power of attorney	50.0

### Consultations on advance directives

**Figure 1.**
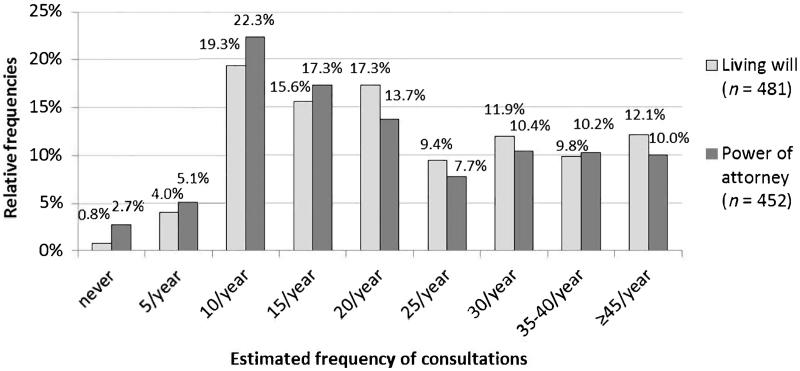
Physician’s estimates on the frequency of consultations on advance directives per year.

More than 96% of GPs stated that they advise patients on preparing ADs: 99.2% perform consultations on LWs and 96.9% on PAs. Most physicians (72.3%) address end-of-life issues in selected patients. More than 20 consultations per year each on LWs and PAs are performed by 60% and 50% of GPs, respectively (see Figure 1).

Consultations on LWs were estimated to last a mean 20.7 min, while those on PAs last 15.7 min. Eighty per cent (80.8%) use predefined templates, mainly from the German Medical Association (43.8%) or the Federal Ministry of Justice (11.5%). A mean of €47.4 was considered an appropriate fee for a 30-min consultation on ADs (SD 34.9; 0–400). See Table 2 for details on consultations.

**Table 2.** Characteristics of consultations on advance directives (*n* = 482).

	Per cent for categorical variables; mean (SD, MINV, MAXV) for continuous variables
Performs consultations on AD (LW and/or PA)	96.9
Performs ≥20 consultations on LWs per year	60.7
≥20 consultations on PA per year	51.7
≥20 consultations on each document (LW and PA) per year	48.6
Mean duration of consultations on LW, min (SD, MINV, MAXV)	20.7 (12.2, 2, 120)
Duration of consultations on PA, min, mean (SD, MINV, MAXV)	15.8 (10.6, 1, 120)
Consultations on LW last ≥20 min	52.3
Consultations on PA last ≥15 min	51.5
GP uses templates for LW	80.8
GP uses templates for PA	71.7
Patients often bring templates for LW	45.0
Patients often bring templates for PA	39.2
GP combines consultations on LW and PA	93.6
Addresses AD in selected patients	72.3
Chronically ill patients	64.1
Pensioners	42.5
Considers AD consultations as easy to integrate in office hours	27.7
Considers fee of ≥€50 appropriate for consultation on AD	24.6
Files LW as scan or paper copy	68.3
Files PA as scan or paper copy	65.7

AD: advance directive; LW: living will, PA: power of attorney; min: minutes; SD: standard deviation; MINV: minimal value; MAXV: maximum value.

### Associations between physician and consultation characteristics

Bivariate analysis showed that GPs were significantly more likely to have their AD documents if they were ≥54 years of age compared to younger ones (55.9% versus 44.1%; *p* = .008), had attended a course on palliative care (56.4% versus 43.6%; *p* = .002), and with additional degree in palliative care (62.2% versus 37.8%; *p* = .021) while there was no association with gender and the duration of consultations (see Table 3).

**Table 3.** Physicians with and without own advance directive: group comparison and regression model.

	GP has own advance directive (*n* = 227) %	Does not have own advance directive (*n* = 228) %	*p *value
Male	50.6	49.4	.763
Age ≥54 years	55.9	44.1	.008
Additional training in palliative medicine:			
Completed course in palliative medicine	56.4	43.6	.002
Holds sub-specialization degree in palliative medicine	62.2	37.8	.021
Completed course or has sub-specialization	56.7	43.3	.002
Consultation on LW ≥20 min	52.7	47.3	.266
Consultation on PA lasts ≥15 min	50.6	49.4	.709
Associations between physician characteristics and having own advance directive (logistic regression model):			
	Odds ratio	95% confidence interval	*p* value
Age ≥54 years	1.775	1.218–2.586	.003
Completed course in palliative medicine	1.669	1.101–2.529	.016
Holds sub-specialization degree in palliative medicine	1.327	0.758–2.323	.322

The logistic regression revealed that a higher age and completion of a course in palliative medicine were the determining factors for having an AD (see Table 3).

Additional bivariate analyses of the relation between GP characteristics and performing ≥20 consultations for LWs as well as PAs per year showed significant associations with the following characteristics: physician has own ADs (58.3% versus 39%; *p* <.001) and has completed one of two trainings in palliative medicine (course completed: 57.8% versus 39.4%, *p* <.001; subspecialty degree: 65.9% versus 45.6%, *p* <.001); there was no association between gender and age. See Table 4 for details.

**Table 4.** Physicians with and without higher consultation frequency (≥ 20 consultations for advance directives per year): group comparison and regression model.

	GP performs ≥20 consultations %	GP performs <20 consultations %	*p *value
Male	50.6	49.4	.267
Age ≥54 years	50.6	49.4	.460
Additional training in palliative medicine:			
Completed course in palliative medicine	57.8	42.2	<.001
Additional certificate in palliative medicine	65.9	34.1	.001
Consultation on LW ≥20 min	48.1	51.9	.781
Consultation on PA lasts ≥15 min	49.4	50.6	.851
Association between physician characteristics and higher consultation frequency (logistic regression model):			
	Odds ratio	95% confidence interval	*p*-value
Male	0.731	0.480–1.111	.143
Age ≥54 years	1.019	0.697–1.490	.921
Has additional training in palliative medicine	1.971	1.347–2.886	<.001
Has own advance directives	2.010	1.376–2.936	<.001

The final statistical model confirmed these results: GPs with own ADs and/or training in palliative medicine were significantly more likely to counsel ≥20 patients per year for each document (see Table 4).

## Discussion

### Main findings

The key issue in population-centred ACP is the question of who advises whom on end-of-life decisions assuring patient’s autonomy and avoiding unwanted and unnecessary medical services. This first survey among German GPs provides initial answers as it shows that nearly all German GPs surveyed counsel patients on LWs and/or PAs. Appropriately, the key target groups addressed by GPs are chronically ill patients. Physicians with own ADs and additional training in palliative medicine counsel significantly more patients, while their counselling time equals that of physicians who counsel fewer patients. These findings warrant a discussion.

### Interpretation in relation to extended literature

First, most GPs are willing to discuss advanced care with their patients. This reflects physicians’ attitude towards end-of-life aspects, although this service is not covered by the statutory health insurance so that some physicians might be prevented from offering those consultations [18]. Given this willingness, one may wonder why the prevalence of AD in Germany is still insufficient. In agreement with other studies from e.g. Denmark and Norway [11,19,20], our survey identified a lack of time during regular office hours as an important barrier for AD counselling: 72.3% of GPs stated that AD consultations are difficult to integrate into regular office hours. Other barriers addressed in prior studies, e.g. a lack of GPs’ knowledge and presumed negative attitudes of patients, were barely mentioned as reasons not to provide advice on ADs [11,19,20].

Furthermore, a wide variation of the duration of consultations was reported (5–120 min). The mean duration of consultations (20.7 min for LW, 15.8 min for PA) may appear rather short considering the 90-min consultation typically considered for ACP by experienced facilitators [3]. Such comparison does not reflect the fact that most GPs have known their patients for many years [21]. They can easily rely on extensive knowledge about the patient, his next-of-kin, the living circumstances including neighbourhood support as well as typically several prior discussions on priorities in healthcare, e.g. in context of the diagnosis of a severe illness or a terminal disease of a family member (e.g. ‘I want to die peacefully like my mother, not with all the tubes like my uncle’). This trust was well documented in prior studies, e.g. the AgeQualiDe study showed that 28.3% of oldest old patients (≥85 years) were not interested in written documents as they believed that their GP and family members would make the right decisions for them [12]. In contrast to these long-standing relationships, external ACP facilitators would need to start from scratch and require considerably more time.

Although it is widely stated that GPs do not counsel enough on ADs, there are no data on what might be appropriate for a typical practice. Surveys showed that about 25–65% of patients are interested in ADs [22,23]. Attempting an estimate, we calculated that a physician with an average-size patient population who counsels 25 patients per year would need about two years to match his patients’ needs (assumptions: average number of patient per GP in three months: 800; 25% > age 70 of whom 60% have chronic diseases; of these, 40% are interested in AD).

Moreover, as shown in a qualitative Swiss study [24], standardized AD templates are useful tools to start conversations about end-of-life preferences. This approach is also chosen by most GPs in our survey, who prefer the shorter template provided by the German Medical Association (43.8%) compared to that of the Federal Ministry of Justice (11.5%). This is in line with a survey among 300 GP patients aged ≥65 years which showed that most people prefer short and easy templates [25].

Given that the statutory health insurance does not cover reimbursement for AD counselling, GPs named a wide range of reimbursement fees which they consider appropriate (€0–400), with the majority naming about €50 for a 30-min consultation, which corresponds to the scale of charges recommended for private patients [18].

In addition, our finding that GPs with own ADs counsel more frequently is an important finding as it reflects a physician’s willingness to actively deal with the personal finitude of life as a relevant factor for patient care.

### Strengths and limitations

The high response rate is a strength of our study. A recall bias (e.g. the duration of consultations) and/or socially desirable answers cannot be excluded. For example, the rate of GPs’ documents could be higher in our study than in the population. As in other studies [26,27], we excluded a selection bias regarding GPs’ age, gender, and practice type by a comparison with national physician data and teaching practices data [26]. A higher rate of palliative training was shown in our population when comparing to all GPs in NRW (17% versus 13.7%) [28], which likely reflects respondents’ interest in the topic, reflecting a selection bias. The lacking reimbursement for this kind of consultation and the gap between the duration of a GP consultation <8 min in Germany could have let increased the estimated duration of 20 min or longer by more than 50% of GPs. Sociable desirable answers should also be considered so that the rate of GPs’ documents could be higher in our study than in the population and the frequency of consultations might be overestimated.

## Conclusion

This first study on German GPs’ counselling on end-of-life issues showed that nearly all GPs surveyed counsel patient issues on end-of-life preferences including the preparation of AD documents but to a variable extent. GPs who had prepared AD for themselves showed higher consultation frequencies on end-of-life issues than those without such provisions.
